# The Relationship Between Mental Health Problems and Systemic Family Dynamics Among High School and University Students in Shaanxi Province, China

**DOI:** 10.3389/ijph.2021.1603988

**Published:** 2021-09-06

**Authors:** Zhe Yang, Yi Cui, Yifan Yang, Yue Wang, Haiyue Zhang, Ying Liang, Yuhai Zhang, Lei Shang

**Affiliations:** ^1^ Department of Health Statistics and Ministry of Education Key Lab of Hazard Assessment and Control in Special Operational Environment, Air Force Medical University, Xi’an, China; ^2^ Equipment Department, Xijing Hospital, Air Force Medical University, Xi’an, China

**Keywords:** systemic family dynamics, mental health, family characteristics, mediation analysis, adolescents

## Abstract

**Objectives:** The present study aimed to correlate relationships between systemic family dynamics and mental health and to explore family factors that influence adolescent mental health in Shaanxi Province, China.

**Methods:** A cross-sectional survey was conducted to sample adolescents aged 12–23 using a questionnaire including Self-rating Scale of Systemic Family Dynamics, Symptom Checklist-90 Revised, and general demographic.

**Results:** More educated parents in white-collar employment and higher family income were associated with better mental health and better family dynamic scores. The total score of family dynamics was positively correlated with mental health scores. The generalized linear mixed model found that poorer mental health was associated with increased age, being in senior high school, having a father in a blue-collar profession, and SSFD square. The structural equation modelling suggested that this is largely a mediated effect *via* those characteristics impacting family dynamics, which in turn affect mental health.

**Conclusion:** Family dynamics may be an important contributor to adolescent mental health. Education and interventions aimed at improving family dynamics may be useful for reducing the prevalence of mental health problems amongst adolescents.

## Introduction

Globally, many children and adolescents experience mental health problems, many of which can interfere with their development and impair daily functions [1]. This issue applies both to developed and developing countries, although there are variations in how relevant data are collected. The World Health Organization reported in 2020 that 10–20% of children and adolescents worldwide experience mental disorders [2]. The World Bank reported in 2006 that for young people, neuropsychiatric disorders are a leading cause of health-related burden, accounting for 15–30% of the disability-adjusted life-years (DALYs) lost during the first three decades of life [3]. The prevalence of adolescent mental health problems also varies in different countries. In the general population, up to 10% of children and 20% of adolescents may meet the criteria for an anxiety disorder at any point in time in the United Kingdom [4, 5]. Furthermore, annually, about 5% of children experience developmental, behavioral, and emotional problems in the United States [6].

In China, there are reasons for concern regarding the mental health of adolescents [7]. One estimate is that the prevalence of total behavioral and emotional problems of children and adolescents enrolled in primary and middle schools across China, aged 6–16 years, was 17.6% in 2020 [8]. Another study found the detection rate of psychological problems among adolescents to be 26.3% in 2019, with the top three issues being learning pressure, compulsion, and anxiety [9].

Adolescence is a complicated critical life stage that bridges childhood to adulthood [2]. Psychological problems of children and adolescents not only affect their academic performance and social interactions, but also contribute to serious problems in adulthood, including unemployment, drug use, and crime [10]. Improving mental health can help teenagers improve their social skills, enhance their ability to solve problems, promote their self-confidence, and avoid violence and other dangerous behaviors [11].

Numerous studies have suggested an association between family characteristics and mental health problems in adolescents. Parental education, family composition, and family economy are correlated with the mental health of adolescents [12]. Furthermore, increased incidence of mental health problems is correlated with poorer family functioning [13, 14]. The autonomy and relatedness in an adolescent’s family are also linked to a range of positive outcomes, such as self-esteem [15]. A large-scale prospective study found that earlier exposure to frequent changes in family dynamics in middle childhood is particularly associated with reduced late-adolescent mental health [16]. In addition, the family environment can contribute significantly to mental health problems, such as depressive symptoms, anxiety, and alcohol abuse [17–19]. Similarly, family satisfaction has a positive effect on psychological well-being [20, 21]. Moreover, in a family environment that lacks security, responsibility, and entertainment, and is full of conflict, young peoples’ difficulties obtaining support and the lack of emotional expression may aggravate their mental health problems [22].

Systemic family dynamics play a key role in the development of an individual personality, the formation of values, the cultivation of social adaptability and physical and mental health [23]. Systemic family dynamics are not only an important measure of familial functionality but are also a fundamental factor that affects healthy physical and psychological child development [24].

However, there are few previous studies that examine the relationships between family dynamics and the mental health of adolescents. This study aimed to elucidate such connections by surveying mental health and family dynamics using standard questionnaires in a large sample of high school and university students in Shaanxi Province, China.

## Methods

### Participants and Sampling

The sample was powered to detect a 2.0% delta at 16.0% prevalence and allowed for a 5.0% non-response rate based on the prevalence of adolescent psychological problems in China [9, 13, 24]. Additionally, considering school and class clustering, the planned sample size was 5,231 and was calculated according to the following parameters:

$$n={\left(\frac{u_{\alpha /2}}{\delta }\right)}^{2}*p*\left(1-p\right)*\left(1+\left(B-1\right)*ICC\right)$$

B is the average cluster size (B = 45) and ICC is the intra class correlation (with an assumed value of 0.065).

From August 2014 to May 2015, a total of 5,435 students received questionnaires, of which 5,188 were validly completed and used in the current analysis. These numbers resulted in an effective recovery rate of 95.5%.

Sampling was performed by sending questionnaires to students in two township junior high schools, one county junior high school, one urban junior high school, two county senior high schools, one urban senior high school, and two universities. In each junior high school (3 years) and senior high school (3 years), four classes for each of the years were selected for a total of 84 classes. At each university, five classes were chosen for all 3 years (30 classes). Questionnaires were distributed to all students present in each class on the day of testing. Students unwilling or unable to participate simply did not complete the questionnaires.

### Survey Instruments

The questionnaire package includes three parts: socio-demographics, the Self-rating Scale of Systemic Family Dynamics (SSFD), and the Symptom Checklist-90-Revised (SCL-90-R). The socio-demographic variables included place of residence, gender, education, family composition, parents’ education, parents’ profession, and family monthly income.

The SCL-90-R is one of the most widely used and well-known self-report instruments designed to measure psychological problems and symptoms of psychopathology, and has been extensively used in Chinese studies. The SCL-90-R consists of 90 items across nine dimensions: somatization (12 items), obsessive-compulsive (10 items), interpersonal sensitivity (9 items), depression (13 items), anxiety (10 items), hostility (6 items), phobic anxiety (7 items), paranoid ideation (6 items), psychoticism (10 items), and others (7 items). The Likert-type question-answer model was used in the SCL-90-R (none = 0, too much = 4) [25–27]. The nine scales of the SCL-90-R are also reliable in adolescents [28]. The mean score of all items in each dimension represents the severity of that mental health issue for each person. Scores > = 2.5 on each dimension were defined as being suggestive of potential mental health problems [29].

The SSFD is the only localized family dynamics scale based on Heidelberg’s family dynamics theory. It was compiled and revised in China [29, 30]. It is self-completed by the individual and thus represents their individual perception of the dynamics of their family. The revised scale has four dimensions, which include 29 items reflecting the perceived dynamics of the family. The four dimensions are family atmosphere (11 questions), personalization (8 questions), system logic (6 questions), and disease concepts (4 questions). The family atmosphere category asks about the emotionality of communication within the family. Questions include the following: can you express strong feelings, can you express care, and can you communicate? The personalization category asks about the free development for family members and questions ask how free members feel regarding their interests, decisions, time spent, and personality. The system logic category asks about the extent to which the family uses “black and white” logic to distinguish items/issues. Questions ask about the extent to which items/issues are judged as right or wrong, good or bad, and very bad or very good. Finally, the disease concepts category asks about family awareness of disease factors. Questions ask about the occurrence of mental illness being related to the family environment, to interpersonal relationships, self-adjustment, and to personal lifestyle. Lower scores on the first three dimensions reflect better family dynamics, as do higher scores on disease concepts.

The scale has good cultural adaptability and reflects the family situation in China comprehensively and systematically. The SSFD has been widely used to assess the characteristics of family dynamics and the changes in family dynamics before and after family treatment [23, 30]. The Likert-type question-answer model was used in the SSFD (completely inconsistent = 0, completely consistent = 4).

The reverse entries were reverse-coded along each dimension before being used to calculate the dimension scores for SCL-90-R and SSFD. The total score was the sum of the scores in each dimension divided by the number of entries, including the disease concept score inverted in SSFD.

### Survey Method

Twenty trained postgraduate students majoring in epidemiology and health statistics were employed as fieldworkers in four groups of five, each with a team leader. During the survey in each class, a head teacher or school counsellor first explained the significance and purpose of the study. Next, the team leader introduced the specific content and completion methods for the questionnaires. During completion, the fieldworkers were available to answer any participant questions. Once the questionnaires were collected, the fieldworker team reviewed them and discarded incomplete or spoiled questionnaires. Participation was voluntary and anonymous. Students unwilling or unable to participate simply remained in class and returned blank questionnaires.

### Statistical Analyses

The means (*M*) and standard deviations (*SD*) are presented for the dimensions and total scores of SCL-90-R and SSFD. Categorical variables are presented as Ns and as percentages of the total study population for the prevalence of mental health problems and general characteristics in adolescents.

Linear mixed models were used to evaluate the differences in the total scores of mental health and family dynamics across categorical socio-demographic variables, adjusted for clustering within schools. Variables that retained independent associations with the outcome at the univariate level (*p*-value < 0.1) were considered for multivariate generalized linear mixed models. The mixed gamma regression models were used to explore which factors were most strongly related to mental health in adolescents. Personal characteristics of adolescents and the total score of family dynamics were taken as fixed effects variables. The gamma regression model with linear and quadratic terms of age and SSFD was run to assess potential non-linear effects, and found the quadratic term of SSFD was statistically significant. A centered version of SSFD including the terms SSFD_cen and SSFD_cen^2 were used in the regression model to make regression coefficient of SSFD more practically meaningful (SSFD_cen = SSFD—mean (SSFD)).

Structural equation modelling was also performed to explore the relationship among basic characteristics, family dynamics, and mental health. Structural equation modelling (SEM) is a multivariate statistical framework that is used to model complex relationships between directly observed and latent variables. Assuming that there were three sections of basic characteristics and that SSFD and SCL-90-R were latent constructs underlying the specific questions, a confirmatory factor analysis (CFA) [31] using SEM [32] was conducted to assess the association between family dynamics and mental health. Maximum likelihood estimation was utilized to estimate the parameters, and several indices were considered to assess the model fit [33]. Bootstrapping was used to assess a potential mediating effect of family dynamics between basic characteristics and mental health. A log-transform of the mental health score was performed before running the SEM-analysis.

All *p*-values are two-tailed. All statistical analyses were performed using IBM SPSS software, version 25 (SPSS© Inc., IBM, Chicago, IL, USA). *p*-values < 0.05 were considered statistically significant.

## Results

The internal consistency and reliability estimates were: SCL-9-R, Cronbach’s α 0.963, split-half reliability coefficient 0.914; SSFD, Cronbach’s α 0.832, split-half reliability coefficient 0.701.

### General Characteristics of the Subjects

As shown in Table 1, SCL-90-R total scores and SSFD total scores differed significantly by urban/rural, parental education level, parental occupation, monthly family income, and educational background. Significantly higher SCL-90-R scores and SSFD scores were both linked to living rurally, having parents with only junior high school education, having blue-collar working parents, and having a family monthly income <3,000 yuan. Participants in senior high school had the highest SCL-90-R scores, while the highest SSFD scores were from junior high school students. Females and students living in a multi-generational family had higher average SSFD-scores than males and students living in a nuclear family, respectively.

**TABLE 1:** Family demographic characteristics of the subjects and Symptom Checklist-90 Revised and SSFD total score comparisons (*n* = 5,188)^a^.

Groups	*n*	%	SSFD score (*M* ± *SD*)	SCL-90-R score (*M* ± *SD*)
Age				
12–14 years	2044	39.4	2.54 ± 0.39	1.47 ± 0.46**
15–17 years	1897	36.6	2.58 ± 0.40	1.64 ± 0.55
>18 years (reference)	1,247	24.0	2.30 ± 0.43	1.43 ± 0.50
Place of Residence				
Urban	2073	40.0	2.39 ± 0.46**	1.45 ± 0.47*
Rural (reference)	3,115	60.0	2.57 ± 0.39	1.57 ± 0.53
Gender				
Male	3,073	59.2	2.48 ± 0.44**	1.49 ± 0.50
Female (reference)	2,115	40.8	2.52 ± 0.40	1.57 ± 0.52
Education level				
Junior high school	2,795	53.9	2.58 ± 0.39**	1.42 ± 0.49**
Senior High school	1,402	27.0	2.52 ± 0.41**	1.56 ± 0.60**
College (reference)	991	19.1	2.22 ± 0.43	1.27 ± 0.40
Family composition				
Nuclear family^b^	1735	33.4	2.39 ± 0.46**	1.46 ± 0.49
Multi-generational family^c^(reference)	3,453	66.6	2.55 ± 0.40	1.55 ± 0.52
Father education level				
Junior high school or below	549	10.6	2.64 ± 0.38**	1.58 ± 0.52**
Senior high school	3,286	63.3	2.52 ± 0.41**	1.55 ± 0.52**
College/university or above (reference)	1,353	26.1	2.37 ± 0.46	1.44 ± 0.48
Mother education level				
Junior high school or below	1,010	19.5	2.60 ± 0.40**	1.61 ± 0.56**
Senior high school	3,053	58.8	2.51 ± 0.41**	1.53 ± 0.51*
College/university or above (reference)	1,125	21.7	2.36 ± 0.46	1.43 ± 0.47
Father profession				
Blue collar	3,440	66.3	2.55 ± 0.40**	1.57 ± 0.53**
White collar (reference)	1748	33.7	2.38 ± 0.45	1.44 ± 0.47
Mother profession				
Blue collar	3,805	73.3	2.55 ± 0.41**	1.56 ± 0.52**
White collar (reference)	1,383	26.7	2.36 ± 0.45	1.43 ± 0.47
Family income				
<3,000 yuan	2,634	50.8	2.58 ± 0.39**	1.59 ± 0.53*
3,000–5,000 yuan	1,684	32.5	2.44 ± 0.43*	1.47 ± 0.49
>5,000 yuan (reference)	870	16.7	2.36 ± 0.47	1.43 ± 0.47

a Adjusted for clustering within schools in simple comparisons.

b Parent and children;

c Parents and children and grandparents.

**p < 0.01, *p < 0.05.

### The Prevalence of Mental Health in Adolescents

Of the study cohort, 20.5% (*n* = 317) scored >=2.5 on at least one of the SCL-90-R subscales, which is a standard criterion for poor mental health. Of these, mild distress (2.5 ≦ factor score <3) had 4.1% prevalence, moderate distress (3≦ factor score ≦4) 1.9% prevalence, and severe distress (factor score >4) 0.1% prevalence. As can be seen in Table 2, the most prevalent five problems were obsessive-compulsive issues (12.1%), interpersonal sensitivity (9.9%), hostility (8.4%), depression (8.3%), and paranoid ideation (8.2%).

**TABLE 2:** The prevalence of mental health problems in adolescents (*n* = 5,188).

Dimension	*M* ± *SD*	Normal		Mild		Moderate		Severe		Positive rate (*%*)
		Score <2.5		2.5≦score<3		3≦score≦4		4 < score		
		*n*	*%*	*n*	*%*	*n*	*%*	*n*	*%*	
Obsessive-compulsive	1.71 ± 0.61	4,563	88.0	377	7.3	231	4.5	17	0.33	12.1
Interpersonal sensitivity	1.58 ± 0.60	4,755	91.7	223	4.3	199	3.8	11	0.21	8.4
Hostility	1.55 ± 0.63	4,671	90.0	265	5.1	234	4.5	18	0.35	10.0
Depression	1.54 ± 0.60	4,759	91.8	237	4.6	178	3.4	14	0.27	8.3
Paranoid ideation	1.50 ± 0.56	4,765	91.9	253	4.9	163	3.1	7	0.13	8.2
Anxiety	1.52 ± 0.58	4,772	92.0	247	4.8	154	3.0	15	0.29	8.0
Phobic anxiety	1.45 ± 0.57	4,823	93.0	196	3.8	158	3.1	11	0.21	7.0
Psychoticism	1.46 ± 0.53	4,848	93.5	198	3.8	139	2.7	3	0.06	6.6
Somatization	1.43 ± 0.52	4,896	94.4	178	3.4	107	2.1	7	0.13	5.6
Total	1.52 ± 0.51	4,871	93.9	213	4.1	98	1.9	6	0.12	6.1

### Generalized Linear Mixed Models of Factors Influencing the SCL-90-R Total Score

The data were modeled and analyzed using the generalized linear mixed models, the results of which are shown in Table 3. In the process of model fitting, it was found that gamma regression exhibited the best fitting effect compared with linear, log linear, and negative binomial model. Gamma regression uses a gamma distribution with a log link, which can be used when the target contains all positive values and is skewed towards larger values. In this model, as summarized in Table 3, the random effects were not statistically significant (*p* = 0.13). Poorer mental health (SCL-90-R total score) was associated with increased age, being in senior high school, and having a father in a blue-collar profession.

**TABLE 3:** Results of a mixed gamma regression model of total Symptom Checklist-90 Revised score with a log link and random effects of schools^a^.

Variables (reference)	Coefficient	SE	*t*	*p*
**Model F = 29.958 *p* < 0.001**				
Education level (College)				
Junior high school	0.148	0.043	3.418	0.001
Senior high school	0.245	0.045	5.427	<0.001
Father profession (White collar)				
Blue collar	0.028	0.014	2.014	0.043
Age	0.014	0.004	3.757	<0.001
SSFD_cen^b^	0.201	0.011	18.737	<0.001
SSFD_cen ^2	0.060	0.016	3.860	<0.001

a The gamma regression model with age and SSFD_cen and SSFD_cen^2 terms were ran to assess potential non-linear effects.

b Centered SSFD-variable: SSFD total score—mean (SSFD total score).

The results also show that there was a strong positive non-linear association between the natural logarithm of SCL-90-R-score and SSFD total score, with a slope of 0.201 (SE = 0.011) at the mean 2.5 of SSFD total score. A positive correlation implies that higher SSFD-scores tend to be associated with higher mental health scores (i.e., with worse mental health).

### Structural Equation Modelling of the SSFD and SCL-90-R

**FIGURE 1:**
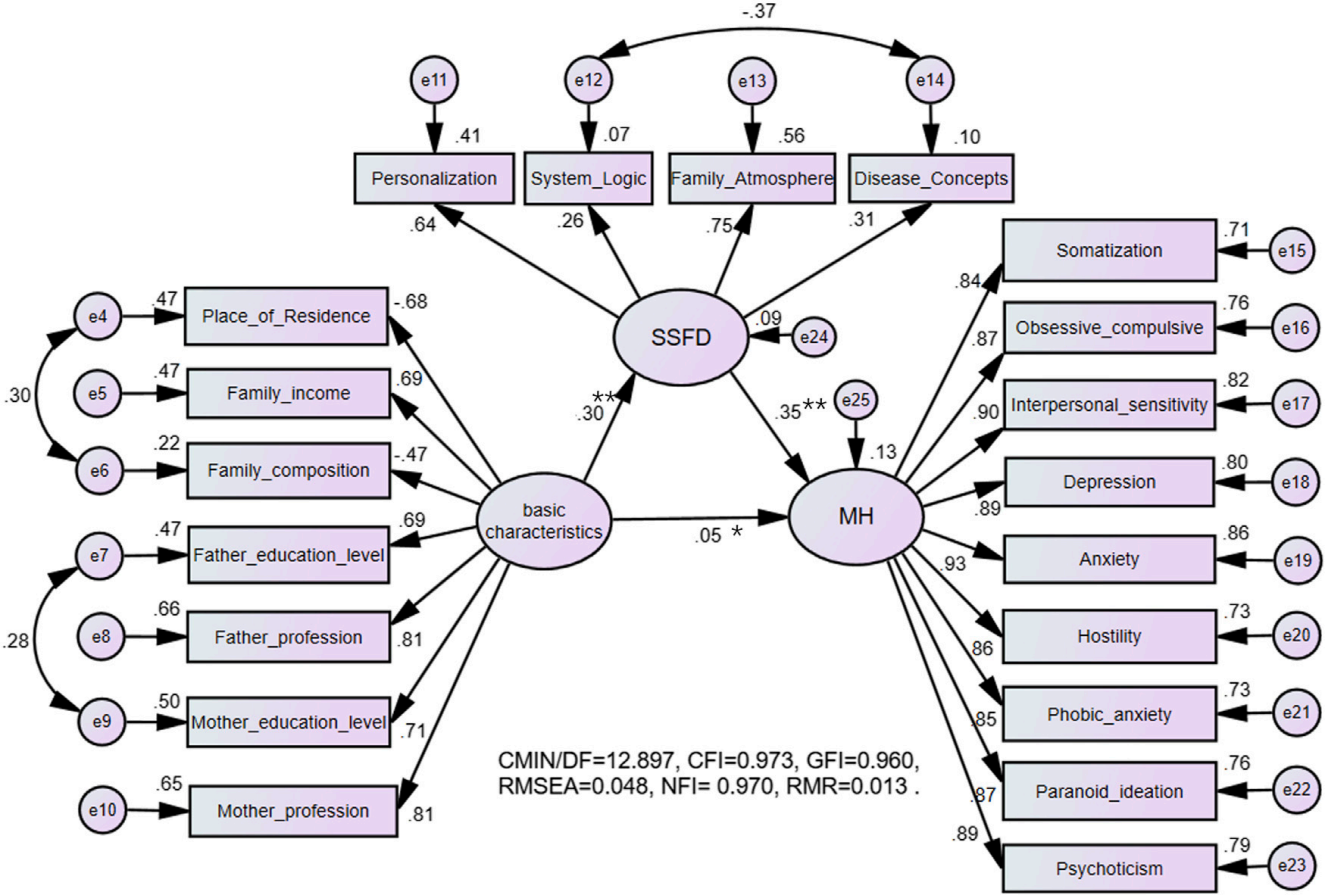
Structural equation modelling of the Self-rating Scale of Systemic Family Dynamics and Symptom Checklist-90 Revised. The name of the study: The Relationship Between Mental Health Problems and Systemic Family Dynamics Among High School and University Students in Shaanxi Province, China; country: Shaanxi Province, China; 2015.

In the process of constructing the SEM, we found that the correlation between age, gender, education level, and latent variables was relatively small (all standardized factor loading <0.1), so we deleted the three observed variables in the model fitting. The results indicate that the robust test statistic performed adequately (see Figure 1). There were relationships between the basic characteristics of the adolescents and their families, family dynamics and mental health, and between family dynamics and mental health (Standardized regression coefficient *β* = 0.30, *β* = 0.05, *β* = 0.35). For basic characteristics, the regression weights were small. The results of SEM show that basic characteristics of the adolescents and their families have a partial intermediate effect on mental health through family dynamics (indirect effect coefficient 0.123, 95CI [0.109–0.140], *p* = 0.006). This implied that basic characteristics of the adolescents and their families indirectly influence mental health through family dynamics (Figure 1). For the explanatory power of the SEM, the basic characteristics and family dynamics accounted for 87% of the variance in the mental health score.

## Discussion

In this survey of Chinese school and college students, family dynamics (SSFD) were related to mental health, as measured by the SCL-90-R. The SCL-90-R subscale scores are consistent with the results of previous Chinese surveys [34].

Sociodemographic variables were also related to mental health. Poorer mental health was associated with living rurally and in a multigenerational family, and also associated with poorer family dynamics. Additionally, having less educated parents in blue-collar employment and a lower family income were also associated with poorer mental health and family dynamics. These findings are consistent with those found previously in various countries [13, 35, 36]. Although rural living was related to poorer mental health, this effect disappeared in generalized linear mixed models after controlling for other sociodemographic variables. This may explain why some studies [37] have failed to find an effect of urban versus rural living. Rural living differs in multiple ways from urban living and, for instance, tends to be confounded with lower parental income and reduced parental educational opportunities.

Senior high school students had poorer mental health than junior high students and college students. This may be due to the college entrance examination, which is crucial for high school students in China and is a source of stress. Additionally, the increasing self-awareness and self-contradiction taking place at that age may play a role [38]. Overall, older students had lower SSFD-scores. The dynamics in the families of these students tend to be better, which might be because as adolescents grow older, they have an enhanced self-awareness and can more easily communicate with family members.

Although mental health differed in standard ways on sociodemographic variables in univariate analyses, some of these variables did not contribute significant variance to the final generalized linear mixed models. Furthermore, the basic characteristics of adolescents have a small and significant effect on mental health, but most of the effects of basic characteristics on mental health were mediated by family dynamics.

These results are also consistent with previous research. A harmonious family atmosphere is related to good mental health [39, 40], while a lack of family recreational activities and poorer communication reduces mental health [41]. Less strict parental control also improves adolescent mental health [42]. Moreover, lack of intimacy and poor expression of emotions in the family can induce adolescent depression [43]. Flexible thinking, indicated by lower systemic logic scores, can reduce psychological problems. This is likely because, when dealing with difficulties, being able to actively think about them and to solve problems using a variety of perspectives has a positive effect on the development of emotions. On the contrary, conventional, uncreative thinking can lead to more emotional behavior problems in teenagers.

This study used a careful sampling strategy that selected convenient educational institutions of three different levels and further structured class sampling within schools to produce a large study cohort to minimize selection bias. Nonetheless, the data were gathered in a single province of China and results may differ elsewhere even though those of the current study are in broad agreement with previous Chinese and global surveys of adolescents. This study has shown that adolescent mental health is related to systemic family dynamics as measured by questionnaires. Limitations of this work include that it was a cross-sectional study and true causality cannot be inferred. Although the survey guaranteed participants privacy and anonymity, some may nonetheless have reported in an unduly positive light. To show that family dynamics influence mental health, it would be necessary to measure family dynamics at time point 1 and then mental health at time point 2. Also, aside from family dynamics and basic sociodemographics, many other factors can affect adolescent mental health. For example, bullying by peers, including cyberbullying, can have profound effects on adolescent mental health. Here, such factors were not assessed.

In the interests of improving adolescent mental health, it would be good practice to set up parental education programs to promote more harmonious family dynamics, advocate for appropriate views of children’s education, build the ability to deal with family conflicts, and address parental psychological problems.

### Conclusion

As is common around the world, this study found that a substantial proportion of adolescents have signs of mental health problems. Interventions to address these problems are required. There is potential to reduce the prevalence of adolescent mental health problems by attending to systemic family dynamics. Ideally, there should be a harmonious family environment and family relationships based on mutual respect, understanding, trust, and care. Parents should also respect their children’s personality, character, and ideas, and provide them with expectations and encouragement as an intimate friend. Moreover, parents should cultivate children’s ability to deal with problems well.
